# Supplementary material for: 6mer seed toxicity in tumor suppressive microRNAs
Source: Nat Commun. 2018 Oct 29;9:4504. doi: 10.1038/s41467-018-06526-1 (PMC6206098; doi:10.1038/s41467-018-06526-1)
Supplement: Supplementary file 1 — Supplementary Information [file 41467_2018_6526_MOESM1_ESM.pdf]

Supplementary Information

6mer seed toxicity in tumor suppressive microRNAs

Gao et al.

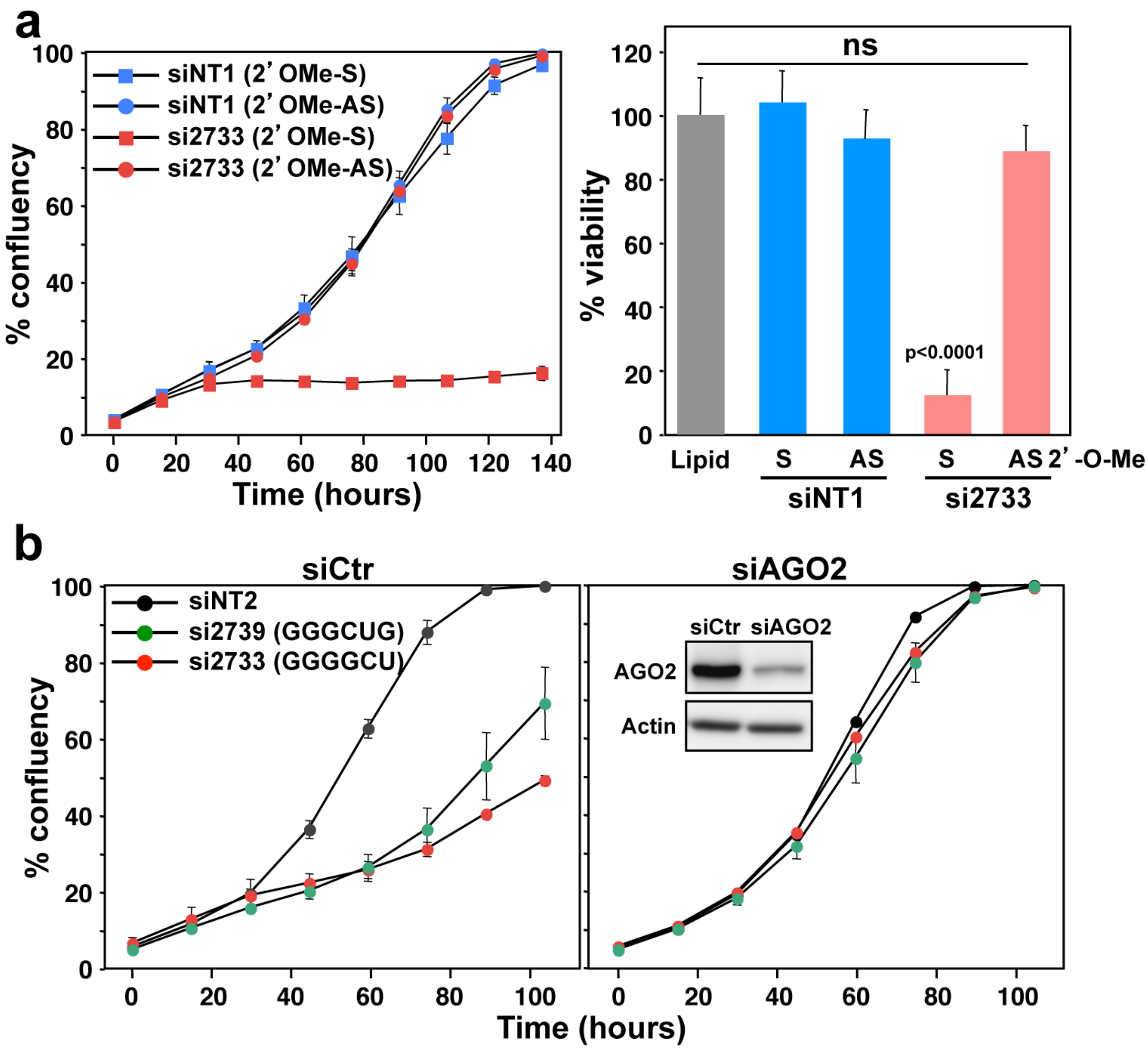

Supplementary Figure 1. Toxic 6mer seeds kill cancer cells through RNAi. (**a**) *Left:* Percent cell confluence over time of HeyA8 cells transfected with 10 nM siNT1 or si2733 with either the guide or the passenger strand modified by 2'-O-methylation in positions 1 and 2. Data are representative of two independent experiments. Each data point represents mean ± SE of six replicates. *Right:* Cell viability 96 hrs after transfecting the cells with the siRNAs shown on the left. Cell viability was determined by quantifying ATP content. Change in cell viability was calculated compared to cells treated with lipid only. p-values were calculated using students ttest. ns, not significant. Data are representative of two independent experiments. Each data point represents mean ± SD of six replicates. (**b**) Percent cell confluence over time of HeyA8 cells transfected with either nontargeting control (siNT2) or the two highly toxic siRNA duplexes (si2739 and si2733) at 10 nM. 6mer seed sequences are given in parentheses. Cells were pre-treated with either nontargeting SMARTpool siRNAs (siCtr, left) or AGO2 SMARTpool siRNAs for 24 hours at 25 nM (right). Insert shows Western blot to document AGO2 knockdown efficiency. Each data point represents mean ± SE of three replicates.

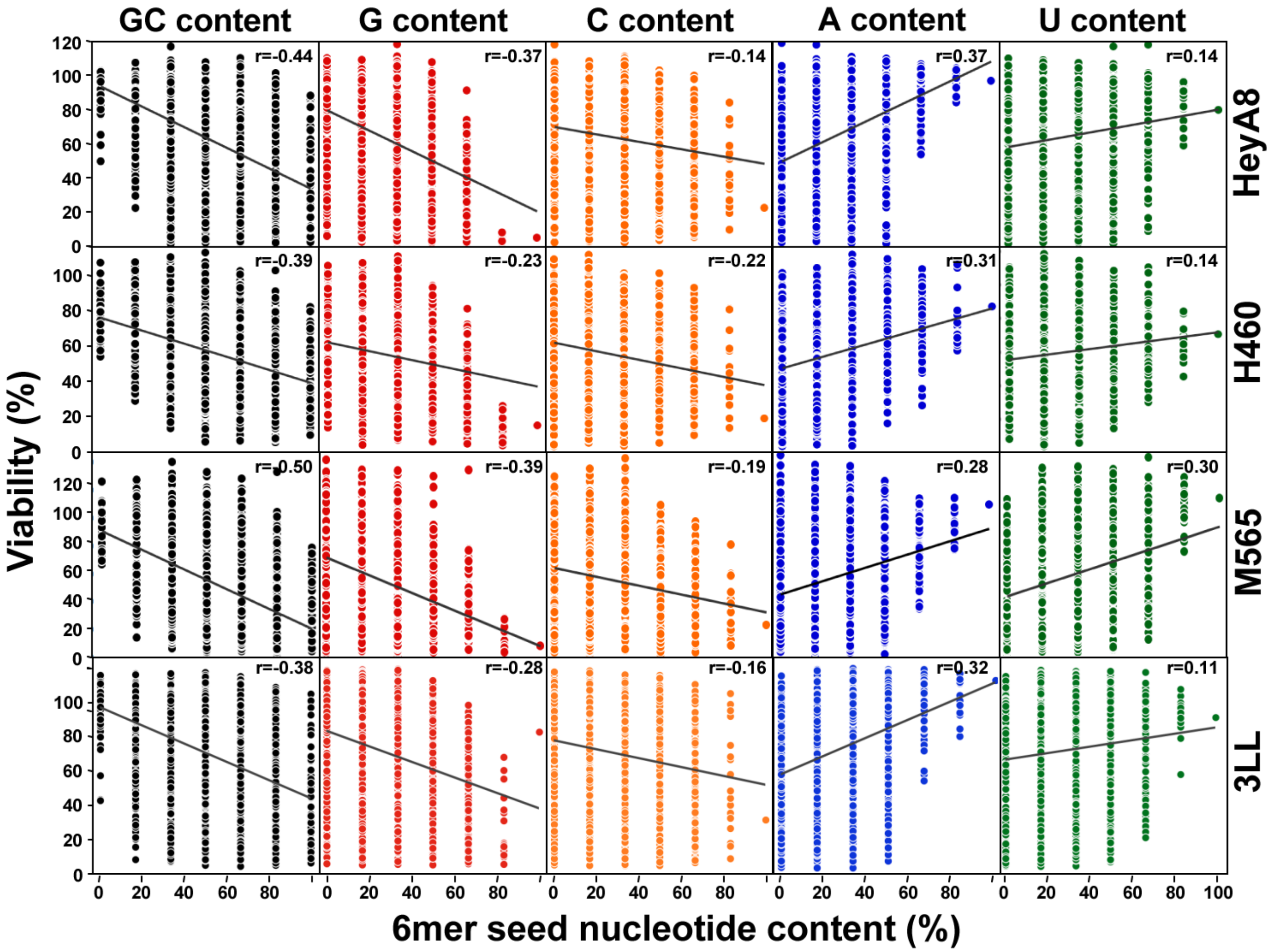

Supplementary Figure 2. 6mer seed toxicity correlates with the nucleotide content of the seed. Regression analysis of cell viability of 6mer seeds in the four cell lines versus GC content or individual nucleotide content (G, C, A, U) of the seeds. All p-values of the Pearson linear correlation coefficients were 0.0.

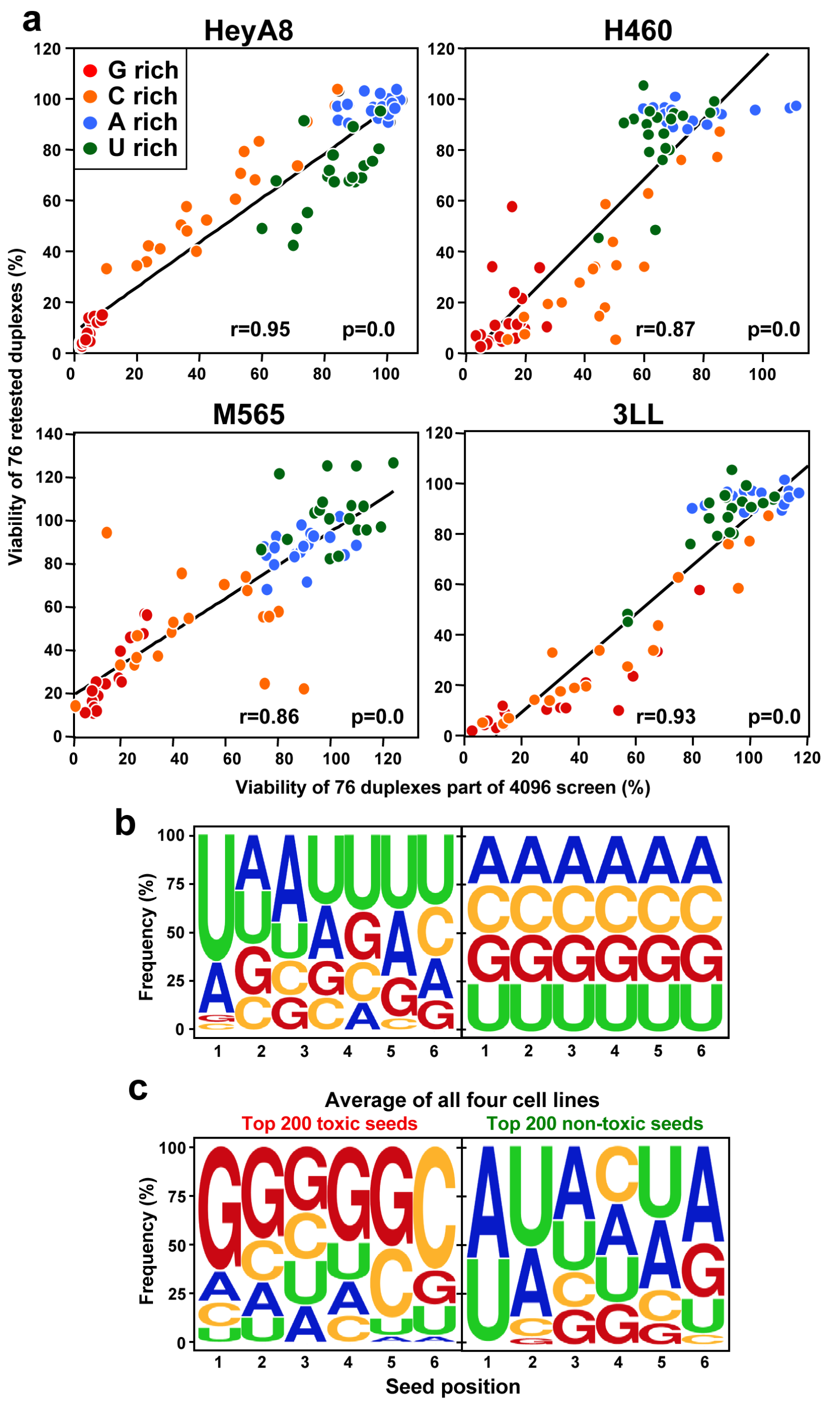

Supplementary Figure 3. Preference for Gs in most toxic siRNAs. (**a**) Regression analysis showing the correlation in cell viability between the original 4096 duplexes screen (x-axis) and the repeat screen with the 76 duplexes with the highest nucleotide content in the seed region in either HeyA8, H460, M565, or 3LL cells. p-values were calculated using Pearson correlation analysis. (**b**) Nucleotide composition at each of the 6 seed positions in a commercial human genome-wide siRNA library (>69,000 siRNAs, left panel) or our balanced 6mer seed duplexes library (right panel). (**c**) Average nucleotide composition at each of the 6 seed positions for the 200 most toxic (left) and 200 least toxic (right) seeds for all four human and mouse cell lines.

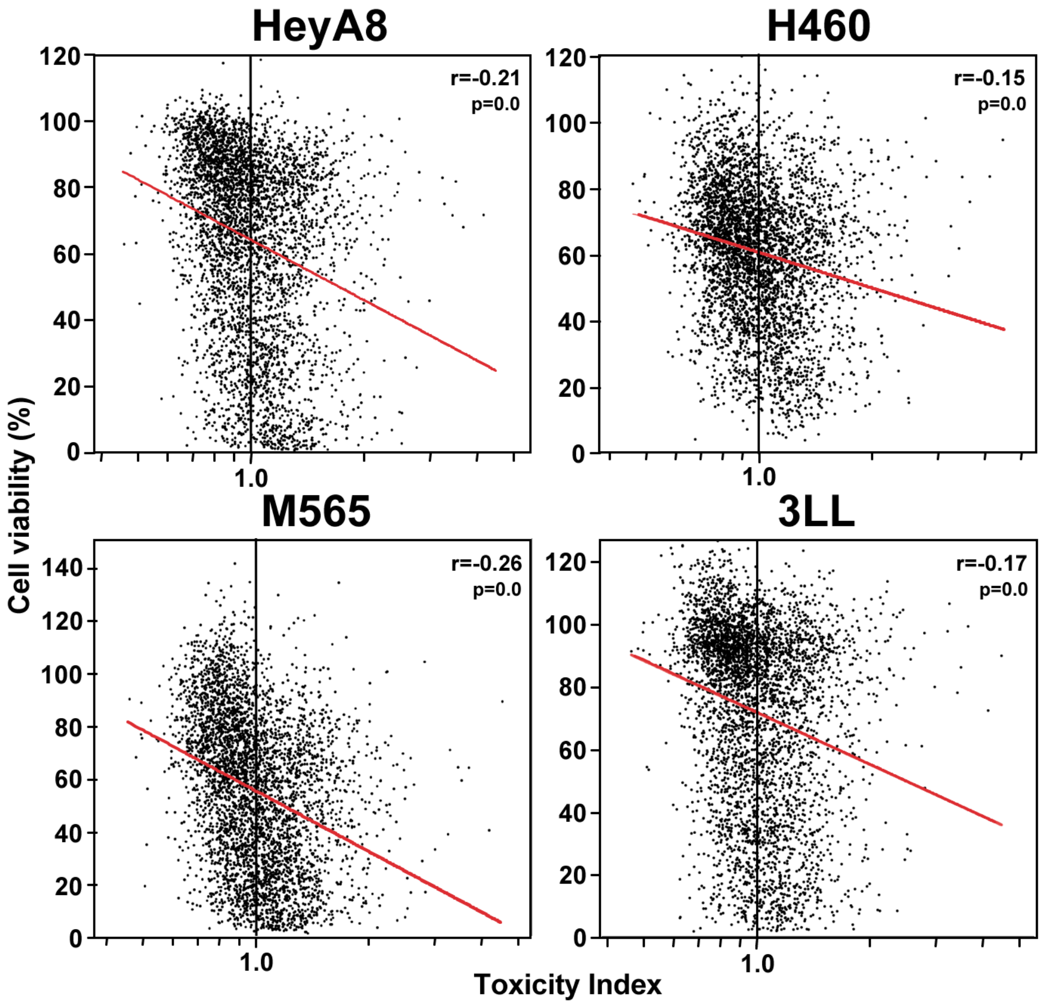

Supplementary Figure 4. The toxicity index (TI) correlates with 6mer seed toxicity. Regression analysis showing the correlation between the cell viability and the 6mer toxicity index (TI) (see [1]) in either HeyA8, H460, M565, or 3LL cells. Note, the data on the two mouse cell lines are an approximation as the original TI analysis was based on SGs and nonSGs identified in lethality screens performed with human cells [1,2]. All p-values of the Pearson linear correlation coefficients were 0.000.

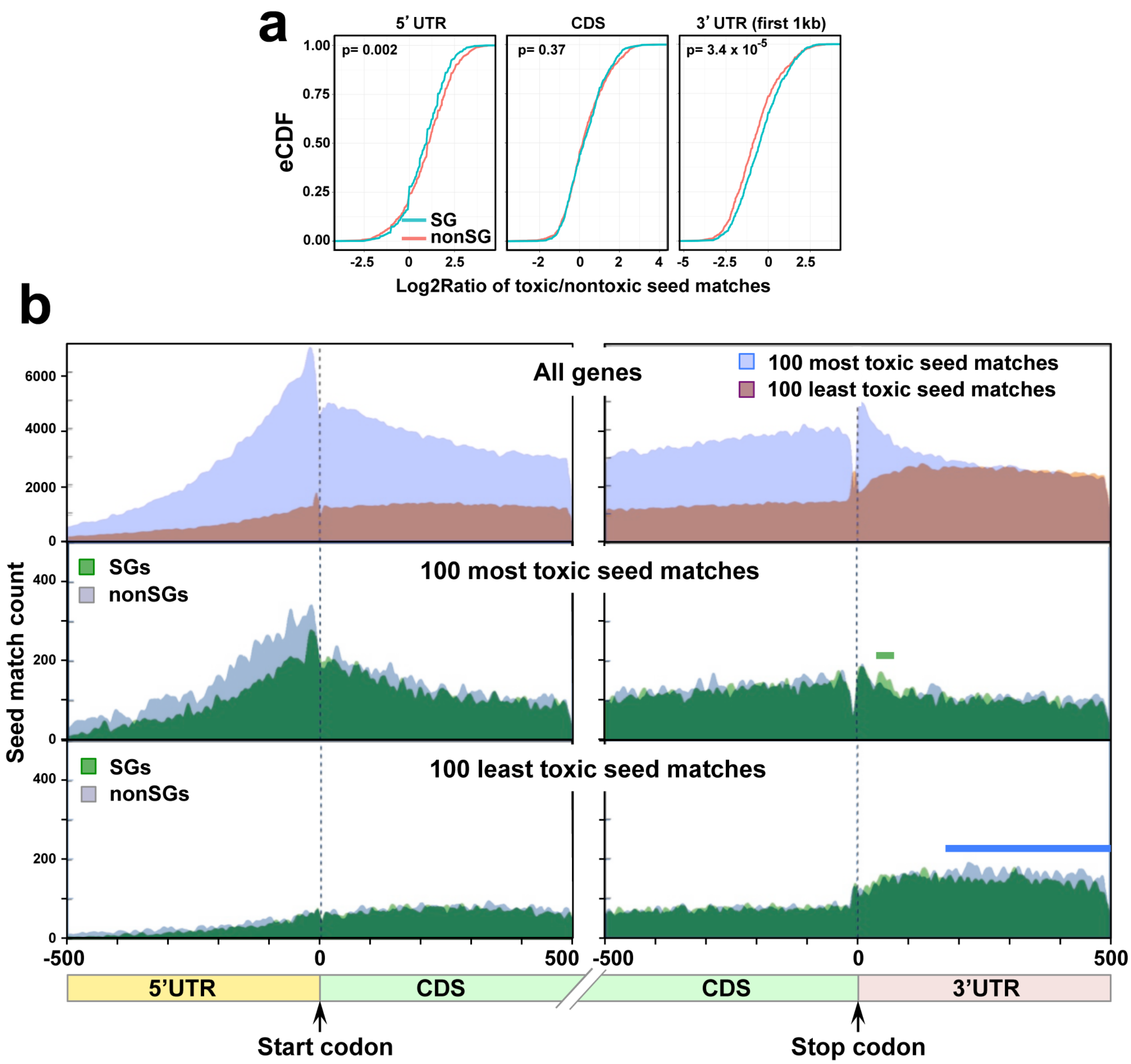

Supplementary Figure 5. Differences in targeting between survival genes and nonsurvival genes by the 100 most and least toxic 6mer seeds. (**a**) eCDF comparing the ratio of occurrences of the 100 most and least toxic 6mer seed matches in the mRNA elements of two sets of expression matched survival genes and nonsurvival genes. p-values were calculated using a two-sample two-sided K-S test. (**b**) Distribution of the seed matches to the 100 most and least toxic 6mer seeds to human cells in human mRNAs around the boundaries of the 5'UTR and the beginning of the CDS and the end of the CDS and the beginning of the 3'UTR (shown are 500 bases in each direction). Data are shown for either all genes (top) or the expression matched SGs and nonSGs (center and bottom). Green horizontal bar, area of enriched toxic seed matches in SGs compared to nonSGs. Blue horizontal bar, area of fewer toxic seed matches in SGs.

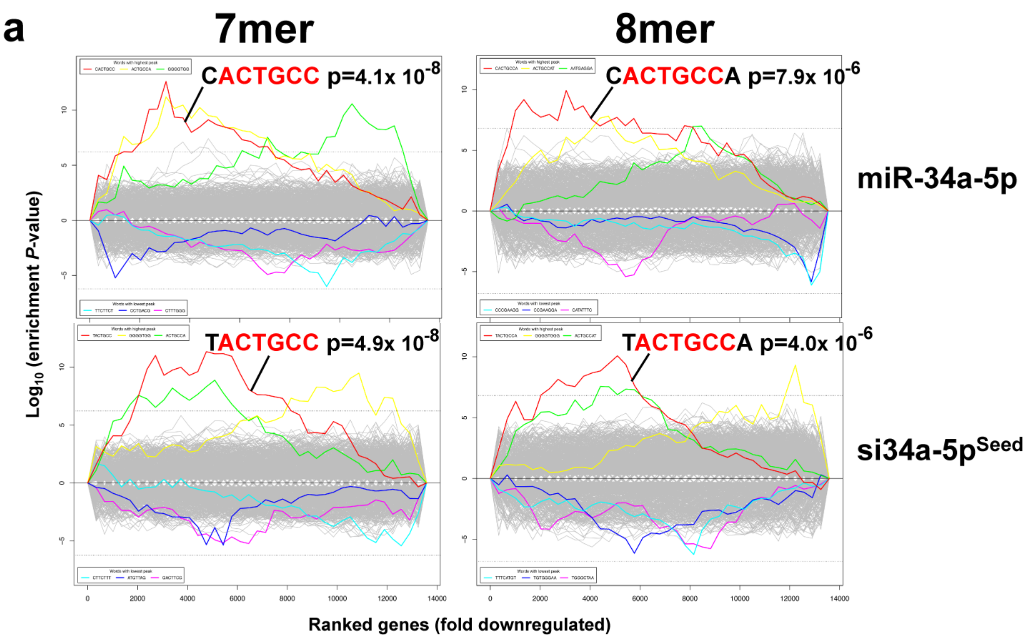

b

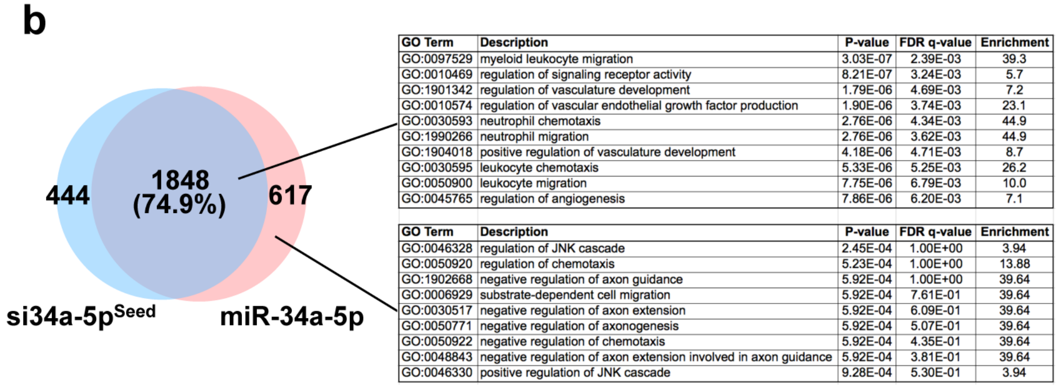

GO Term	Description	P-value	FDR q-value	Enrichment
GO:0097529	myeloid leukocyte migration	3.03E-07	2.39E-03	39.3
GO:0010469	regulation of signaling receptor activity	8.21E-07	3.24E-03	5.7
GO:1901342	regulation of vasculature development	1.79E-06	4.69E-03	7.2
GO:0010574	regulation of vascular endothelial growth factor production	1.90E-06	3.74E-03	23.1
GO:0030593	neutrophil chemotaxis	2.76E-06	4.34E-03	44.9
GO:1990266	neutrophil migration	2.76E-06	3.62E-03	44.9
GO:1904018	positive regulation of vasculature development	4.18E-06	4.71E-03	8.7
GO:0030595	leukocyte chemotaxis	5.33E-06	5.25E-03	26.2
GO:0050900	leukocyte migration	7.75E-06	6.79E-03	10.0
GO:0045765	regulation of angiogenesis	7.86E-06	6.20E-03	7.1

GO Term	Description	P-value	FDR q-value	Enrichment
GO:0046328	regulation of JNK cascade	2.45E-04	1.00E+00	3.94
GO:0050920	regulation of chemotaxis	5.23E-04	1.00E+00	13.88
GO:1902668	negative regulation of axon guidance	5.92E-04	1.00E+00	39.64
GO:0006929	substrate-dependent cell migration	5.92E-04	7.61E-01	39.64
GO:0030517	negative regulation of axon extension	5.92E-04	6.09E-01	39.64
GO:0050771	negative regulation of axonogenesis	5.92E-04	5.07E-01	39.64
GO:0050922	negative regulation of chemotaxis	5.92E-04	4.35E-01	39.64
GO:0048843	negative regulation of axon extension involved in axon guidance	5.92E-04	3.81E-01	39.64
GO:0046330	positive regulation of JNK cascade	9.28E-04	5.30E-01	3.94

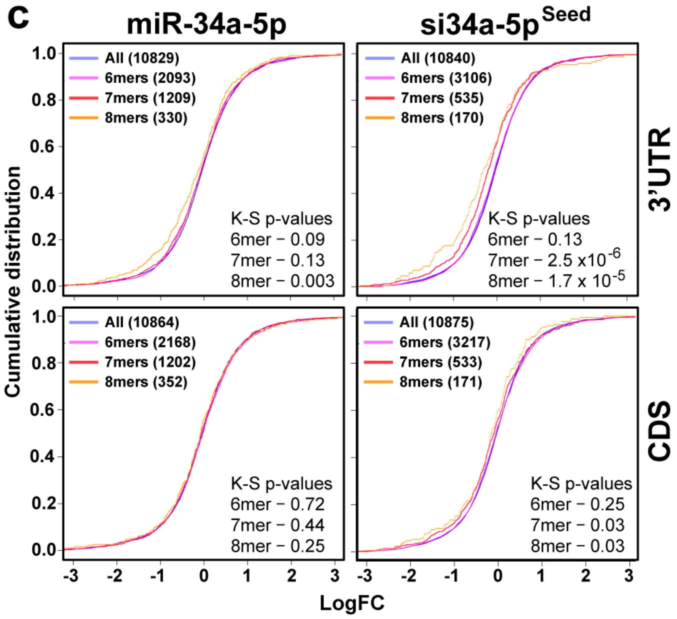

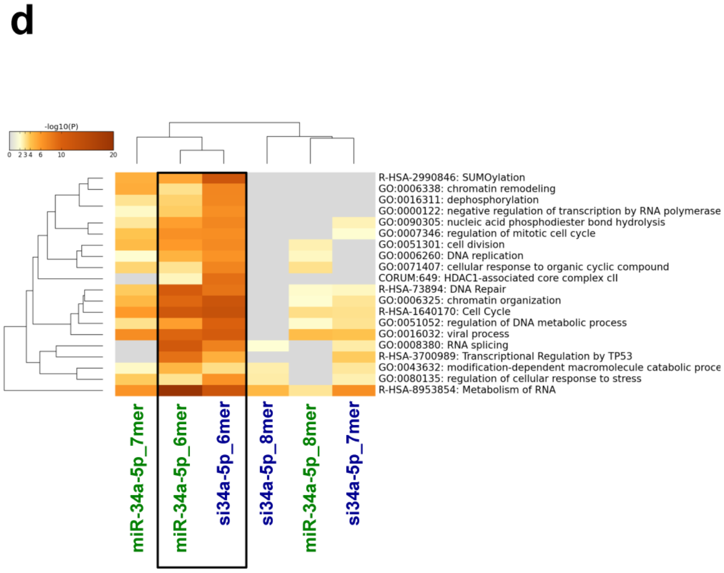

Supplementary Figure 6. Similarities in the effects on cells transfected with miR-34a-5p and si34a-5p. (**a**) Sylamer analysis (7mers and 8mers) for the list of 3‘UTRs of mRNAs in cells treated with either miR-34a-5p (top) or si34-5p^{Seed} (bottom) sorted from down-regulated to up-regulated. Bonferroni-adjusted p-values are

shown. (**b**) Overlap of RNAs detected by RNA-Seq upregulated in HeyA8 cells (>1.5 fold) 48 hrs after transfection with either si34a-5p^{Seed} or miR-34a-5p when compared to either siNT2 or a nontargeting pre-miR, respectively. *Right*: Results of a GOrilla gene ontology analysis of the genes upregulated in both cells transfected with miR-34a-5p or si34a-5p^{Seed} (top, significance of enrichment $<10^{-6}$), or only in cells transfected with miR-34a-5p (bottom, significance of enrichment $<10^{-4}$). (**c**) eCDF of all mRNAs and mRNAs containing a unique 6mer, 7mer or 8mer in their 3'UTR. Only unique 6, 7 and 8mer seed matches were counted. P-values were calculated using K-S test. (**d**) Metascape gene ontology analysis comparing the downregulated genes in cells treated with either miR-34a-5p or si34a-5p^{Seed} containing either unique 6mers, 7mers or 8mers of the respective si/miRNA. GO terms shared by genes downregulated in cells transfected with either siRNA and carrying a unique (not overlapping with either a 7 or 8mer) 6mer are boxed. Note, the downregulated genes that contained unique 7 or 8mers showed no overlap between cells transfected with miR-34-5p or si34a-5p.

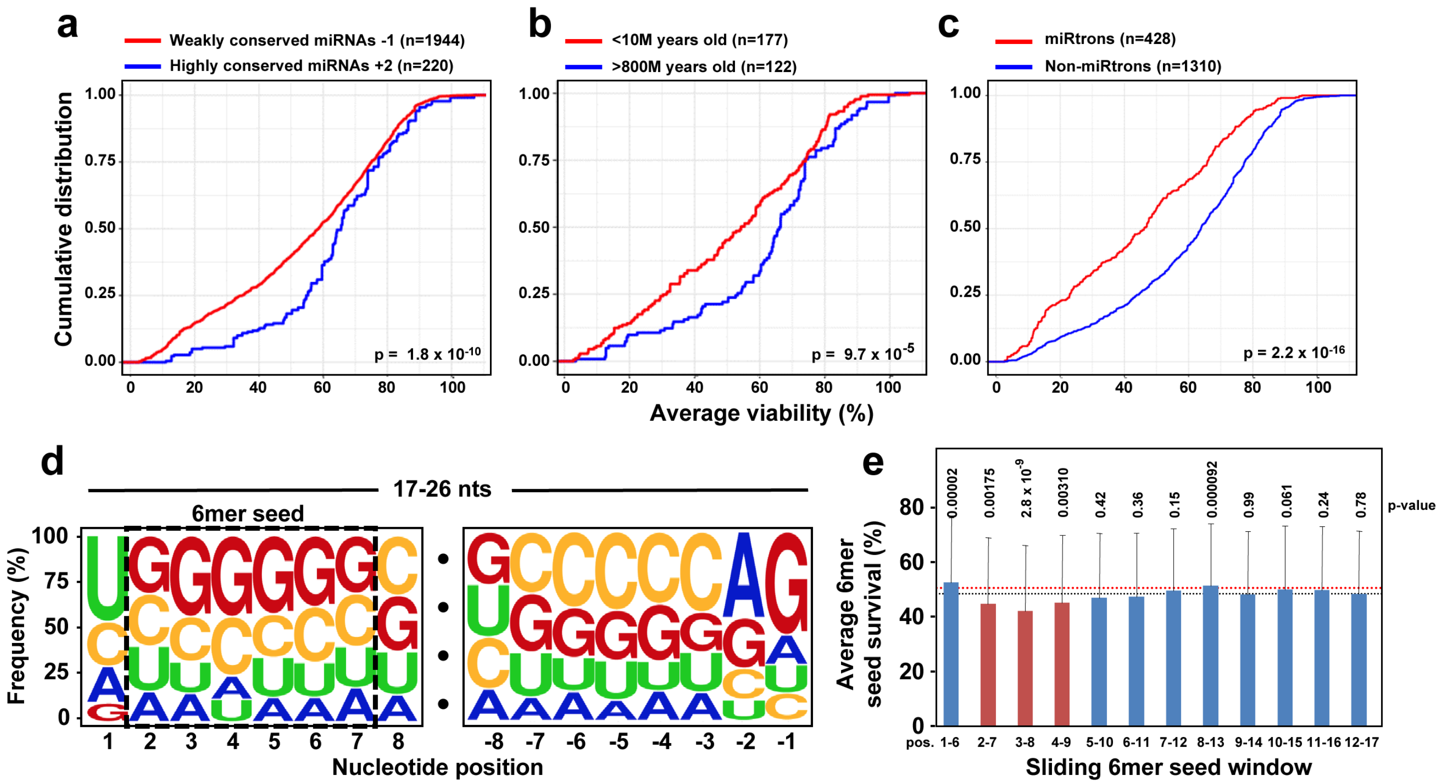

Supplementary Figure 7. Loss of toxic 6mer seeds in miRNAs during evolution. (**a-c**) Data of **Figs. 5a**, left, **5a**, right and **5c** represented as Empirical Cumulative Density Fraction plots. P-values were calculated using K-S test. (**d**) Average nucleotide composition across the 428 mature miRtrons analyzed in **Fig. 6c** (3p and 5p arms) in positions 1-8 at 5' end (left) and the 8 positions at the 3' end (right). The position of the 6mer seed is boxed in a stippled line. (**e**) Predicted average 6mer seed toxicity of 6mer sequences in the first 17 nts of all 428 miRtrons when moved along the first 17 nts of all miRtrons shown as mean ± SD. Red stippled line: 50% viability threshold used to identify toxic seeds in the screens. Red columns indicate seed positions for which the predicated average toxicity was significantly higher than the average across all positions (black stippled line). P-values were calculated using Wilcoxon matched pairs test by comparing the average seed toxicity for each seed position with the average 6mer seed toxicity of all 6mers across all miRtrons (black stippled line).

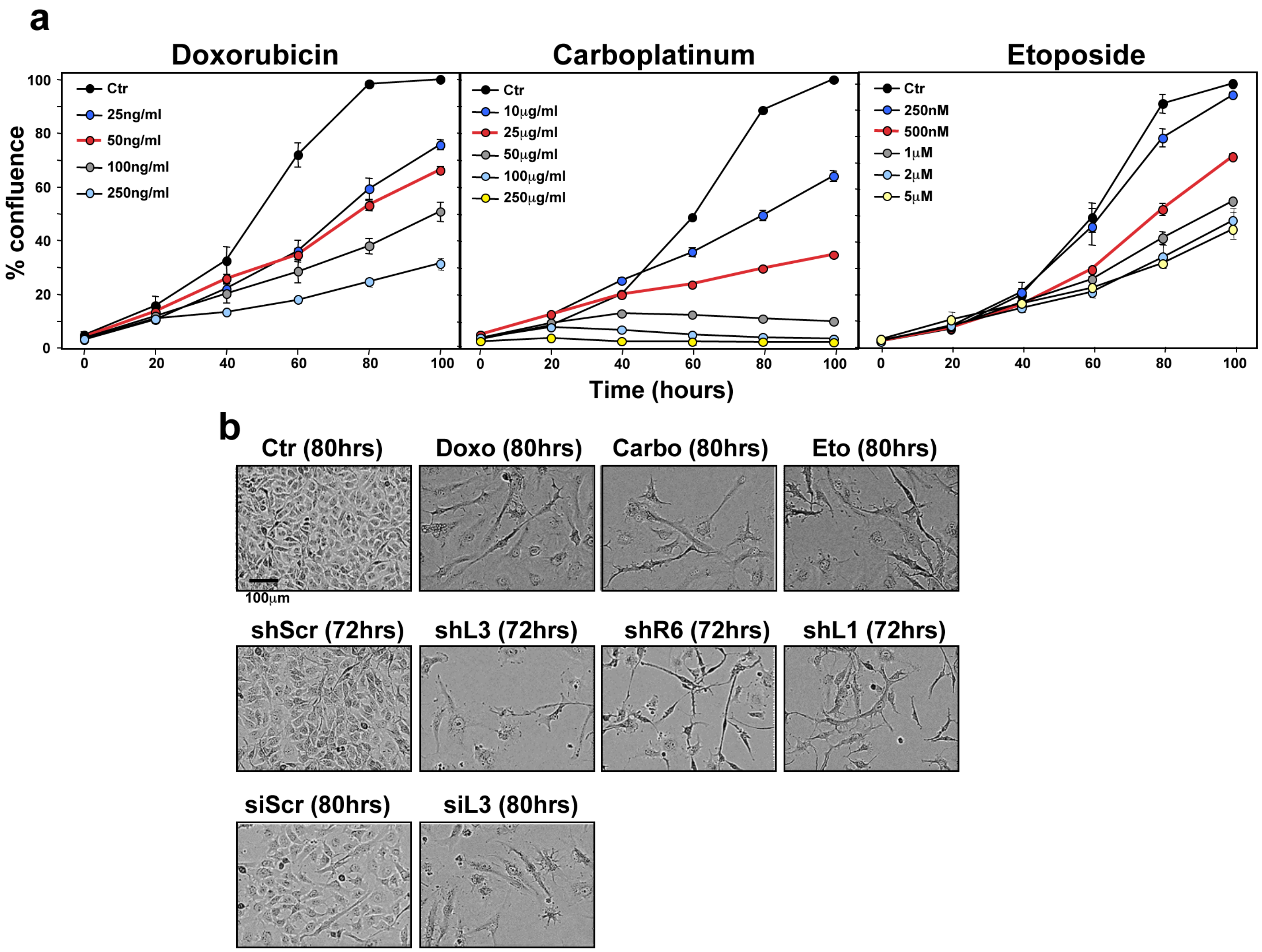

Supplementary Figure 8. Cells treated with genotoxic drugs have features of cells treated with si/shRNAs carrying toxic 6mer seeds. (**a**) Titration of the three genotoxic drugs. Percent cell confluence of HeyA8 cells after treatment with three genotoxic drugs at various concentrations. Medium treated cells were used as control for Carbo and Doxo treated cells. Solvent control treated cells (0.025% DMSO in medium) were used as control for Eto treated cells. Data are representative of three independent experiments. Each data point represents mean ± SE of three replicates. For each treatment the concentration that resulted in the red curve was used for the RNA Seq experiment described in **Supplementary Figure 9**. (**b**) Morphological features of HeyA8 cells treated with three genotoxic drugs or control, with the 3 indicated shRNAs (and shScr), or with 10 nM siL3 and siScr for the indicated time.

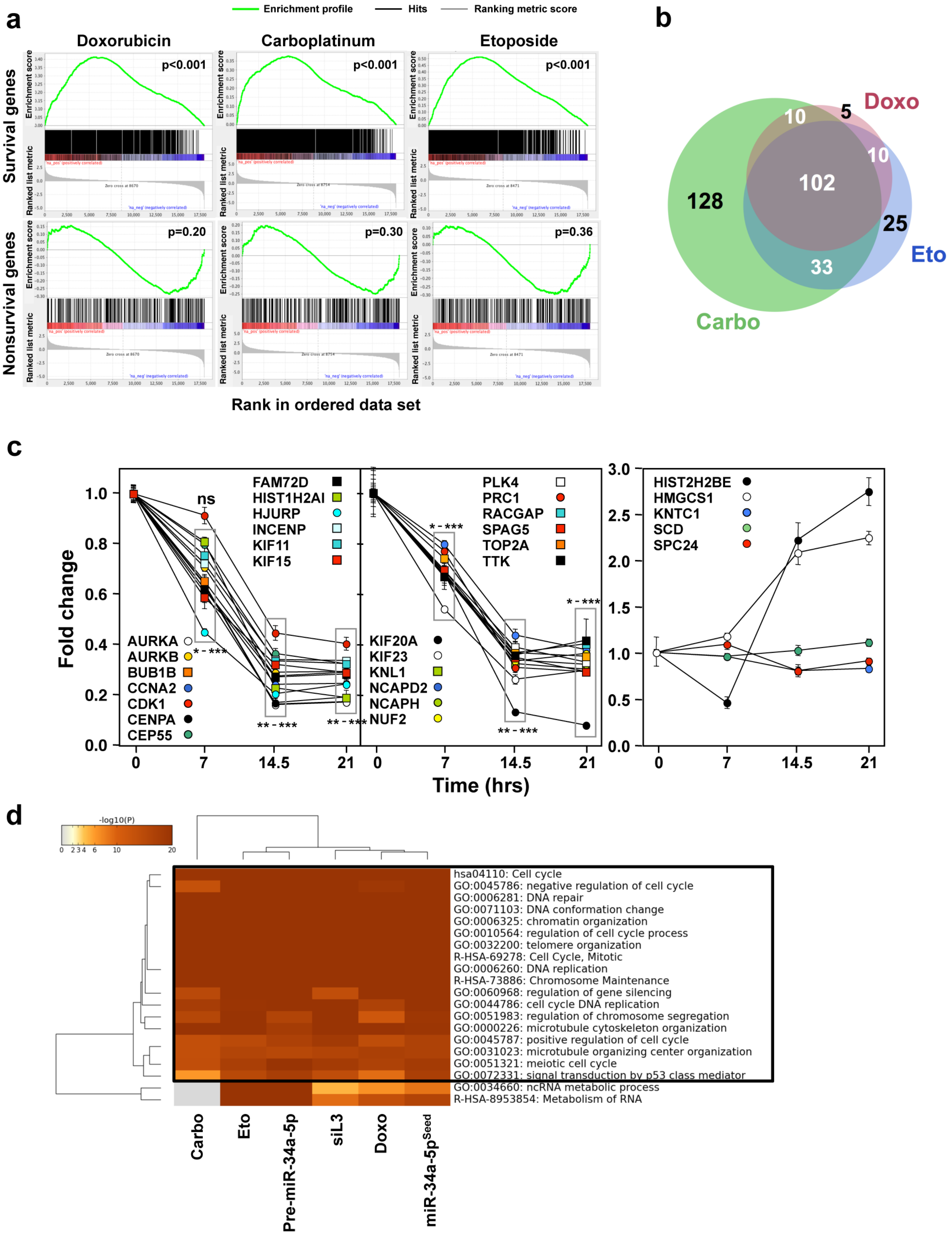
a
Enrichment profile
Hits
Ranking metric score
Doxorubicin
Carboplatinum
Etoposide
Survival genes
Nonsurvival genes
p<0.001
p=0.20
p=0.30
p=0.36
Enrichment score
Ranked list metric
Rank in ordered data set
b
Doxo
Eto
Carbo
128
10
5
10
102
25
33
c
Fold change
Time (hrs)
ns
FAM72D
HIST1H2AI
HJURP
INCENP
KIF11
KIF15
AURKA
AURKB
BUB1B
CCNA2
CDK1
CENPA
CEP55
PLK4
PRC1
RACGAP
SPAG5
TOP2A
TTK
KIF20A
KIF23
KNL1
NCAPD2
NCAPH
NUF2
HIST2H2BE
HMGCS1
KNTC1
SCD
SPC24
d
-log10(P)
hsa04110: Cell cycle
GO:0045786: negative regulation of cell cycle
GO:0006281: DNA repair
GO:0071103: DNA conformation change
GO:0006325: chromatin organization
GO:0010564: regulation of cell cycle process
GO:0032200: telomere organization
R-HSA-69278: Cell Cycle, Mitotic
GO:0006260: DNA replication
R-HSA-73886: Chromosome Maintenance
GO:0060968: regulation of gene silencing
GO:0044786: cell cycle DNA replication
GO:0051983: regulation of chromosome segregation
GO:0000226: microtubule cytoskeleton organization
GO:0045787: positive regulation of cell cycle
GO:0031023: microtubule organizing center organization
GO:0051321: meiotic cell cycle
GO:0072331: signal transduction by p53 class mediator
GO:0034660: ncRNA metabolic process
R-HSA-8953854: Metabolism of RNA
Carbo
Eto
Pre-miR-34a-5p
siL3
Doxo
miR-34a-5pSeed

Supplementary Figure 9. Similarities between genes downregulated after cell death induction with CD95L and CD95 derived si/shRNAs and cell death induced by genotoxic drugs. (**a**) GSEA analysis showing that ~1800 survival genes are preferentially downregulated in HeyA8 cells treated with all three genotoxic drugs. GSEA analysis using a control set of ~400 genes that are not required for survival (as described in [1]) were not enriched in the downregulated genes. p-values indicate the significance of enrichment. (**b**) Venn diagram showing that 102 downregulated survival genes (out of ~1800) (>2 fold, adj p value <0.05) are shared among HeyA8 cells treated with three different genotoxic drugs. A DAVID gene ontology analysis showed that these 102 genes are mostly involved in mitosis and cell cycle (**Supplementary Data File 5**). (**c**) 30 of the 102 survival genes in downregulated in all three genotoxic drugs (see b) were chosen for a kinetics study by real-time PCR. 25 of the 30 genes were found to be substantially downregulated at 14.5 hours. 24 of the 25 genes were significantly downregulated as early as 7 hours after Doxo treatment. Five of the 30 genes were found to be either unchanged or up regulated after Doxo treatment. Each data point represents mean ± SD of three replicates. The 20 hr time point was independently repeated. Significance was calculated using a two-sided t-test. *p<0.05, **p<0.001, *** p<0.0001. (**d**) Metascape gene ontology analysis comparing the downregulated genes in cells treated with either siL3, siR-34a-5p^{Seed}, pre-miR34a-5p or the three genotoxic drugs. GO terms shared by all treatments are boxed.

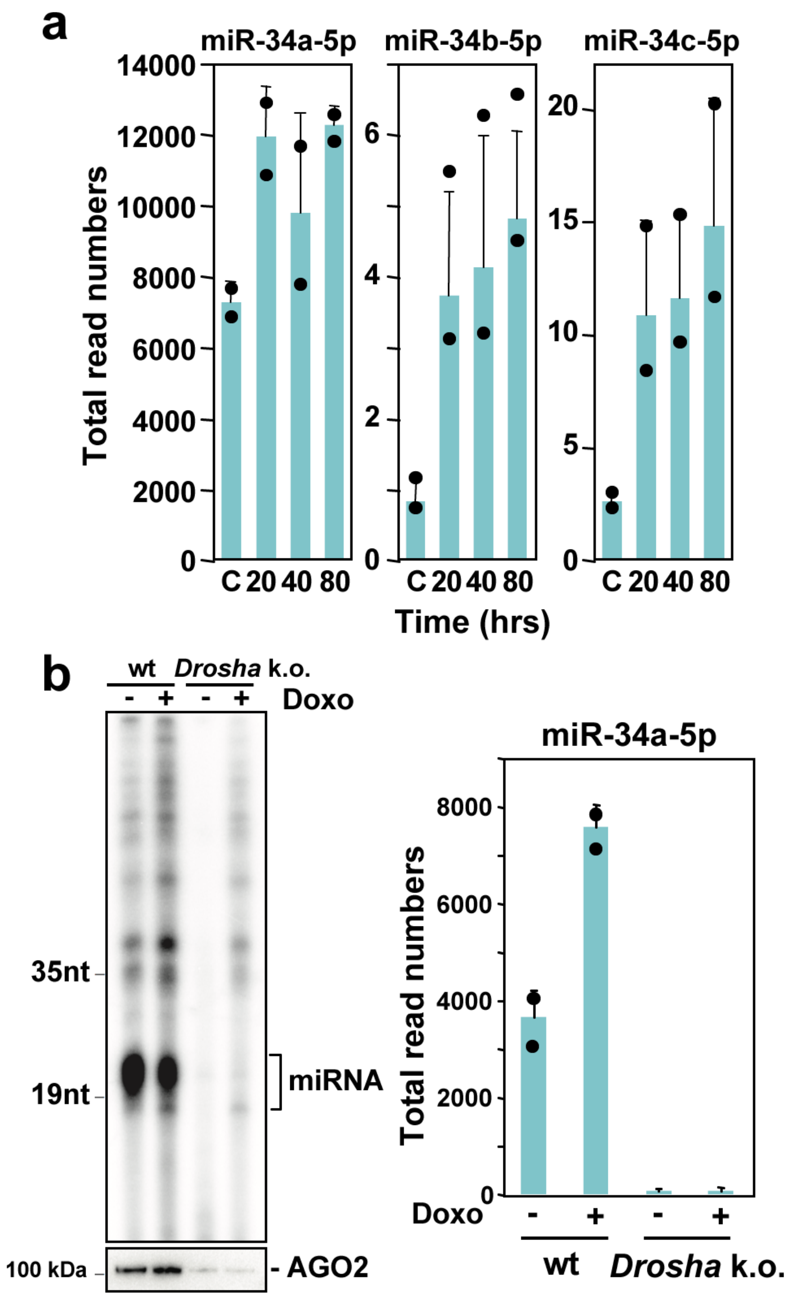

Supplementary Figure 10. Upregulation of Ago-bound miR-34a-5p in cells treated with genotoxic drugs. (**a**) Total read numbers of Ago bound miR-34a-5p (left panel), miR-34b-5p (middle panel), and miR-34c-5p (right panel) in control treated cells or after 20, 40, or 80 hours of Doxo treatment. Shown are individual data points (black dots) and the variance of two biological replicates. (**b**) Ago pull down of miRNAs from HCT116 wild-type and *Drosha* k.o. cells treated with Doxo for 48 hrs (left). The images are representative of two biological replicates. Comparison of read numbers of pre-miR-34a-5p bound to Ago proteins between control and Doxo treated samples in wild-type (wt) or *Drosha* k.o. cells (right). Shown is variance of two biological replicates.

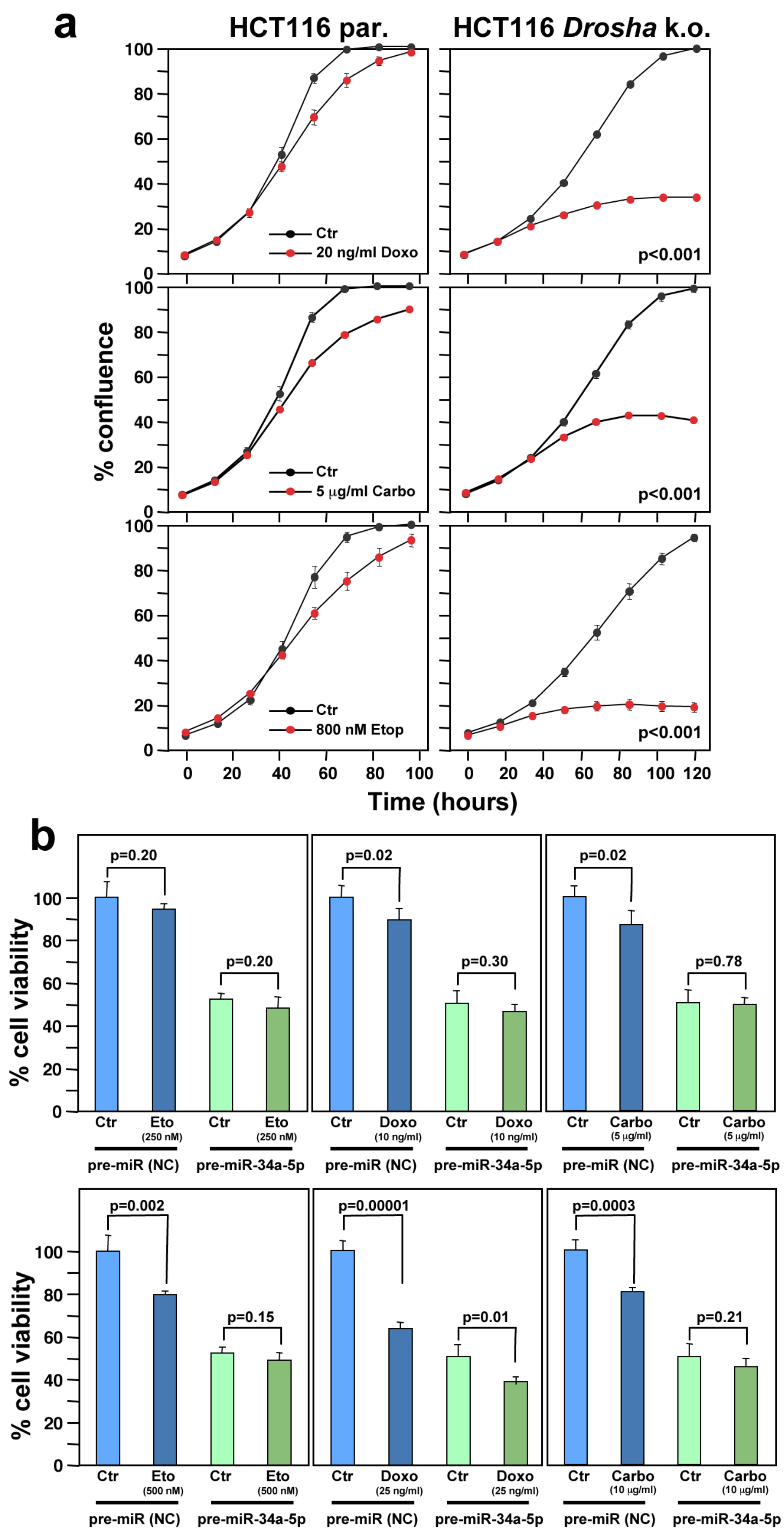
a
HCT116 par.
HCT116 Drosha k.o.
% confluence
Ctr
20 ng/ml Doxo
5 μg/ml Carbo
800 nM Etop
p<0.001
Time (hours)
b
% cell viability
p=0.20
p=0.02
p=0.30
p=0.78
p=0.002
p=0.15
p=0.00001
p=0.01
p=0.0003
p=0.21
Ctr
Eto (250 nM)
Doxo (10 ng/ml)
Carbo (5 μg/ml)
Eto (500 nM)
Doxo (25 ng/ml)
Carbo (10 μg/ml)
pre-miR (NC)
pre-miR-34a-5p

Supplementary Figure 11. Genotoxic drugs act in part through 6mer seed toxicity. (**a**) Percent cell confluence over time of HCT116 parental or *Drosha* k.o. cells treated with three genotoxic drugs. P-values were calculated using a binomial distribution test and support a difference in sensitivity between parental HCT116 and *Drosha* k.o. HCT116 cells. Data are representative of two independent experiments. Each data point represents mean ± SE of three replicates. (**b**) HeyA8 cells were reverse transfected with 1 nM of either pre-miR (NC) or pre-miR-34a-5p. 24 hrs after transfection, NC or pre-miR-34a-5p transfected cells were treated with the genotoxic drugs at two concentrations (very low [top] or low, [bottom]). Cell viability was determined 72 hours after adding genotoxic drugs. The experiment was performed in triplicate. Mean ± SD is shown and p-values were calculated using two-sided t-test.

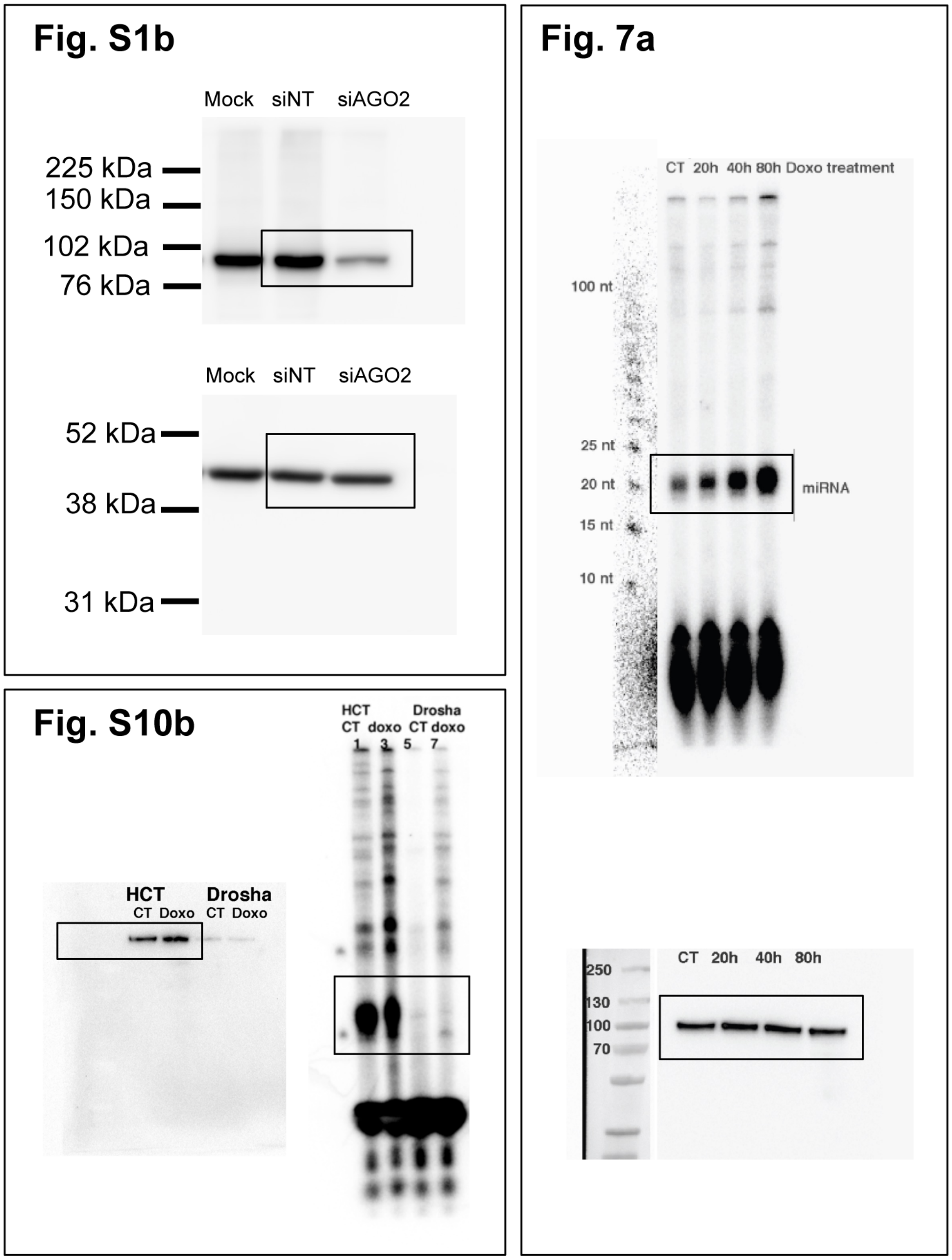

Supplementary Figure 12. Uncropped Western blots and autoradiographs.

Supplementary References

1. Putzbach W*, et al.* Many si/shRNAs can kill cancer cells by targeting multiple survival genes through an off-target mechanism. *eLife* **6**, e29702 (2017).

2. Wang T*, et al.* Identification and characterization of essential genes in the human genome. *Science* **350**, 1096-1101 (2015).
