# Supplementary material for: 6mer seed toxicity in tumor suppressive microRNAs
Source: Nat Commun. 2018 Oct 29;9:4504. doi: 10.1038/s41467-018-06526-1 (PMC6206098; doi:10.1038/s41467-018-06526-1)
Supplement: Supplementary file 3 — Description of Additional Supplementary Files [file 41467_2018_6526_MOESM3_ESM.pdf]

Description of Additional Supplementary Files

File Name: Supplementary Data 1 Description: Results of the 4096 6mer seed toxicity screen in four cell lines. The top 200 most toxic and bottom 200 least toxic duplexes (for each cell line) are highlighted in red and green, respectively. All data are available at https://6merdb.org.

File Name: Supplementary Data 2 Description: The 20 and 100 most and least toxic 6mers to both HeyA8 and H460 cells. Sequences of the most toxic and least toxic 6mers that were used to perform analyses in Fig. 3 and Supplementary Figure 5.

File Name: Supplementary Data 3 Description: Results of RNA Seq analysis of HeyA8 cells transfected with either miR-34a-5p or si34a-5p^{Seed}. Number of genes >1.5 fold downregulated in cells treated with si34a-5p^{Seed} = 2616; Number of genes >1.5 fold downregulated in cells treated with pre-miR-34a-5p = 2868.

File Name: Supplementary Data 4 Description: Ratio of 6mer seed toxicity of the guide strand versus the lesser expressed arm of all miRNAs.

File Name: Supplementary Data 5 Description: Downregulated genes in HeyA8 cells treated with either Doxo, Carbo, or Eto and results of DAVID gene ontology analysis of the 102 genes downregulated in HeyA8 cells treated with the three drugs.

Tab 1: RNA Seq analysis of HeyA8 cells treated with three genotoxic drugs for 80 hrs.

Tab 2: Survival gene (SG) and non-survival gene (nonSG) mRNAs downregulated at least 2 fold with padj of <0.05 in the three treatments.

Tab 3: DAVID analysis of the 102 SGs downregulated in all three treatments.

File Name: Supplementary Data 6 Description: Lists of 938 expression-matched SGs and nonSGs.
